# An empirical model of electronic portal imager response implemented within a commercial treatment planning system for verification of intensity‐modulated radiation therapy fields

**DOI:** 10.1120/jacmp.v9i4.2807

**Published:** 2008-11-11

**Authors:** Rao F.H. Khan, Orest Z. Ostapiak, Joe J. Szabo

**Affiliations:** ^1^ Department of Medical Physics Tom Baker Cancer Center Calgary Alberta; ^2^ Department of Medical Physics Juravinski Cancer Center Hamilton Ontario Canada

**Keywords:** quality assurance, portal dosimetry, amorphous silicon electronic portal imaging device, relative dose verification

## Abstract

Quality assurance (QA) of an intensity‐modulated radiation therapy (IMRT) plan is more complex than that of a conventional plan. To improve the efficiency of QA, electronic portal imaging devices (EPIDs) can be used. The major objective of the present work was to use a commercial treatment planning system to model EPID response for the purpose of pre‐treatment IMRT dose verification.

Images were acquired with an amorphous silicon flat panel portal imager (aS500: Varian Medical Systems, Palo Alto, CA) directly irradiated with a 6‐MV photon beam from a Clinac 21EX linear accelerator (Varian Medical Systems). Portal images were acquired for a variety of rectangular fields, from which profiles and relative output factors were extracted. A dedicated machine model was created using the physics tools of the Pinnacle^3^ (Philips Medical Systems, Madison, WI) treatment planning system to model the data. Starting with the known photon spectrum and assuming an effective depth of 7 cm, machine model parameters were adjusted to best fit measured profile and output factors. The machine parameters of a second model, which assumed a 0.8 MeV monoenergetic photon spectrum and an effective depth in water of 3 cm, were also optimized. The second EPID machine model was used to calculate planar dose maps of simple geometric IMRT fields as well as a 9‐field IMRT plan developed for clinical trials credentialing purposes.

The choice of energy and depth for an EPID machine model influenced the best achievable fit of the optimized machine model to the measured data. When both energy and depth were reduced by a significant amount, a better overall fit was achieved. In either case, the secondary source size and strength could be adjusted to give reasonable agreement with measured data. The gamma evaluation method was used to compare planar dose maps calculated using the second EPID machine model with the EPID images of small IMRT fields. In each case, more than 95% of points fell within 3% of the maximum dose or 3 mm distance to agreement. These results are slightly poorer than those obtained using an ion chamber array, which confirms agreement to within 2% of the maximum dose or 2 mm distance to agreement for all points within these fields.

PACS numbers: 87.55Qr, 87.56.Fc

## I. INTRODUCTION

Recent improvements in the contrast and resolution of electronic portal imaging devices (EPIDs) have made them an increasingly important component of current medical linear accelerators. In addition to their use for patient positioning, significant efforts have been made by various groups to use EPIDs for dose measurement for the purpose of quality assurance (QA) of treatment plans,^(^ ^1^ ^–^ ^4^ ^)^ in vivo dosimetry,^(5)^ ^,^ ^(6)^ positioning verification for multileaf collimator compensator thickness,^(7)^ and in situ dosimetry.^(8)^ Modern amorphous silicon EPIDs are typically characterized by ease of set up, 12‐ to 16‐bit grayscale depth, and submillimeter pixel separation. Prototype versions of amorphous silicon EPIDs have been developed for the measurement of commissioning data from a linear accelerator.^(9)^

Since these devices became available, serious efforts have been made to extract accurate dosimetric information from them. For a commercial EPID, the signal produced in the hydrogenated amorphous silicon matrix has been found to be stable and approximately proportional to dose.^(^ ^10^ ^–^ ^13^ ^)^ But because EPIDs were primarily designed for localization imaging, several problems must be overcome to facilitate dose measurement.

First, the response of the EPID with field size is not water‐equivalent, which poses a problem for direct dose conversion. This problem has been extensively studied and accounted for by several investigators by either measuring or modeling the scatter kernel and glare kernel of the EPID.^(^ ^12^ ^–^ ^15^ ^)^ An EPID's pixel elements are known to over‐respond to low‐energy photons.^(^ ^16^ ^–^ ^18^ ^)^ This over‐response is attributed to enhanced photoelectric interaction of low‐energy photons in the high atomic number phosphor layer, resulting in a 13% increase in response relative to central axis at 15 cm off‐axis where the beam has a greater low‐energy component. This effect also reduces the EPID response to radiation transmitted through closed multileaf collimator (MLC) leaves relative to an open beam by a factor of 1.28 at central axis because of beam hardening through the MLC.^(19)^

Second, backscatter from components of the EPID support arm downstream from the detector have been found to influence the signal by up to 5%.^(20)^ ^,^ ^(21)^

Third, a major problem arises from the automatic accounting of differential pixel response of the EPID. Individual raw pixels of the aS500 show differences in sensitivity. An auto‐correction process involves the division of raw images by the flood field (FF) image. This FF image is acquired with a large open field, and all raw EPID images are divided by it before they are stored and displayed. However, the division procedure overlooks the fact that an inherent beam profile is present in both the raw image and the FF image and is therefore eliminated from the final stored image. Furthermore, pixels in various regions of the FF may see a different photon spectrum, which influences their relative response correction through their non‐water‐equivalent response. Both effects result in a corruption of dosimetric response of the EPID. Various approaches are used to overcome this problem: for example, physically altering the FF to be uniform,^(8)^ ^,^ ^(11)^ ^,^ ^(21)^ or correcting the EPID image using a measured dose profile.^(3)^ ^,^ ^(22)^ A fluence map deduced from the EPID image can later be convolved with dose deposition kernels for a patient or water phantom, thereby predicting the planar dose inside the patient or water phantom.^(15)^ ^,^ ^(23)^ ^,^ ^(24)^ Greer^(8)^ presented a method to measure off‐axis response and to subsequently account for it by further processing.

A further issue pertains to the ghosting effects in the portal imager, which result from the change in pixel sensitivity and image lag in the readout of signal. These effects arise from the fact that residual charge may be trapped in the bulk silicon matrix, possibly altering the electromagnetic field and hence the sensitivity of a pixel.^(25)^ ^,^ ^(26)^

In the present paper, we introduce a novel practical approach to IMRT plan verification. We define EPID response as the auto‐FF and dark field–corrected planar image arising from accumulation of charge in pixel elements because of direct irradiation of the portal imager at a given source–to–portal imager distance without the addition of an absorber in the beam. The decision to forgo the buildup layer was made for the following reasons: the setup is simple, the EPID images appear sharper, and no modifications to maintenance and EPID QA procedures are needed. Our approach involves using the physics tools of a commercial treatment planning system (Pinnacle^3^: Philips Medical Systems, Madison, WI) to create a dedicated machine model that, for a given source‐to‐surface distance (SSD) and assumed effective depth in water, yields the planar dose distribution at the isocenter of an IMRT beam that matches the image acquired by the EPID centered on the isocenter irradiated by the beam at the same SSD. The machine model parameters—energy, incident fluence profile, and secondary scatter source width and intensity—were adjusted to best fit calculated profiles and output factors to those extracted from EPID images.

The model parameters of the dedicated EPID machine may not necessarily be regarded as having physical meaning, but the ability to fine‐tune them at the commissioning stage to achieve the best possible fit with measurements is of great benefit to a QA analysis because the detection limit of errors is minimized (in contrast to most other methods published in literature, which model the physical EPID in a physical beam spectrum in an effort to predict response). Furthermore, to simplify its practical implementation, this technique models the response of the EPID used without additional buildup and with default imaging settings. Most of the modeling approaches are based on physical Monte Carlo modeling of the EPID or on empirical measurements.^(22)^ ^,^ ^(27)^ The work presented here is focused on the modeling of EPID response using convolution–superposition‐based treatment planning software in a manner similar to that seen in the work of Van Esch et al.,^(3)^ except that here, the existing water convolution kernels were used, whereas in the work of Van Esch, they were replaced with the EPID response function. The work of Van Esch and colleagues also reports on the effects of ghosting and imager saturation. These effects are equally relevant to our work, but are not reported here. We report the development of an empirical EPID machine model in Pinnacle^3^ and the testing of the model against EPID images of IMRT test patterns and of fields from a clinical trials phantom benchmark plan.

## II. MATERIALS AND METHODS

### A. Measurements for EPID commissioning

All measurements were performed using an amorphous silicon aS500 EPID (Varian Medical Systems, Palo Alto, CA) supported by the Exact Arm (Varian Medical Systems) centered on the machine isocenter. A 300 monitor units (MUs) per minute 6‐MV photon beam was used for irradiation. The EPID was operated in IMRT mode (0 syn delay, 0 reset frames, 9996 frames average) using Portal Vision client software ver. 7.3.10, IAS2 software ver. 6.1.11, and a model IDU‐11 detector. In this mode, the EPID performs signal readout from individual pixels frame by frame until the beam is turned off. A frame is a single readout of the whole matrix of 512×384 pixels. The integrated IMRT acquisition mode results in a single image that is the sum of all frames acquired during beam delivery, divided by the number of frames. Portal images were acquired for various field sizes defined by jaws and MLCs (Table 1). The data were processed using ImageJ, a Java‐based public domain image processing software application developed at the National Institutes of Health (http://rsb.info.nih.gov/ij/index.html). In‐plane and cross‐plane profiles through the central axis were both extracted from the image. From the image of a given field, the central axis response is determined from the mean grayscale value of the central 9×9 pixels. Averaging over this (approximately) 7‐mm square reduces the effect of pixel sensitivity variation. Output factor (OF) was calculated as the ratio of mean grayscale for a given field to that of a 10 cm square reference field.

**Table 1:** Fields used for modeling electronic portal imaging device (EPID) response in Pinnacle^3^, expressed as a single dimension for square shapes or as width by length for rectangular shapes

*Jaws (cm at isocenter)*	*MLC (cm at isocenter)*
1, 2, 3, 5, 8, 10, 15, 18, 20, 25, 28	Parked
10	1, 2, 3, 5, 6, 8
	6×1,6×2,6×3,4×3,4×2,3×6,3×4,3×2,3×1,2×6,2×4,2×3,2×1,1×6,1×3,1×2
20	10,15,18,18×10,15×6,15×3,10×18,10×4,6×15,4×18,4×10,3×5 10 (offset 5 cm cross‐plane)

### B. Pinnacle modeling

A Pinnacle^3^ (ver. 7.4f) radiation therapy planning system was used to model the EPID response. The convolution–superposition algorithm used in Pinnacle^3^ is based on the work of Mackie et al.^(28)^ ^,^ ^(29)^ and Papanikolaou et al.^(30)^ For incident energy fluence, the algorithm calculates total energy released per unit mass (TERMA) in the medium, which is subsequently convolved with the energy deposition kernel to compute dose in the medium.^(5)^ Using ImageJ, in‐plane and cross‐plane profiles and OFs were extracted from EPID images of the field geometries set out in Table 1. The profiles were then imported into Pinnacle^3^ with the depth in water set to 7 cm. (Once the EPID profiles are imported, the assigned depth in water is not a parameter that can be varied.) The choice of 7 cm as the depth was based on observations of our own and those of others^(12)^ that variation in EPID central pixel response as a function of field size approximates that of an ion chamber at between 5 cm and 10 cm depth in water. Preserving this variation in the model is important if IMRT segments of various sizes are to sum with correct relative magnitude.

The clinical machine model of a Varian 21EX 6‐MV photon beam was used as a starting point for this model (henceforth called PinEPID7). The adjustable model parameters were energy spectrum, radial fluence profile, primary source size, secondary source size and strength, jaw and MLC transmission, and electron contamination. In contrast to the work of Van Esch et al.,^(3)^ our work with the physics tools within Pinnacle^3^ did not provide the ability to replace the scatter kernel with the dose response function for the EPID. The clinical beam photon spectrum was retained for the PinEPID7 model. All other model parameters were iteratively adjusted until a best fit with measured profiles and output factors was achieved. A second model (PinEPID3) was created in which the water‐equivalent depth of the measured EPID profiles was taken to be 3 cm while preserving the same source‐to‐detector distance of 100 cm. In this geometry, a clinical beam photon spectrum does not model the observed variation of EPID response with field size. At shallow depths, this variation is better fitted using lower photon energy. A monoenergetic beam was used because the model must reproduce the EPID response only at the single fixed depth of irradiation.

**Figure 1:**
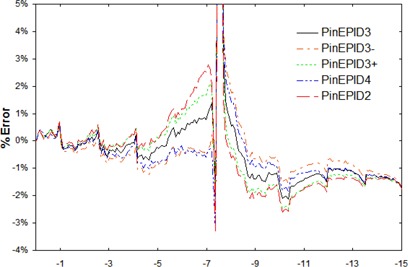
Percent error in computed minus measured profiles normalized to central axis plotted as a function of off‐axis distance (in cm). The curves correspond to the PinEPID3 model and the models derived from it by altering either the modeling depth (PinEPID2 and PinEPID4) or machine energy (PinEPID3+ and PinEPID3–) as explained in the text. The dose error peaks to about 15% at the field edge because of the large dose gradient there. Nevertheless, the distance to agreement in this neighborhood is within about 2 mm.

**Figure 2:**
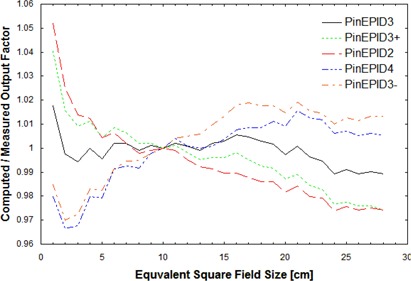
Ratios of computed‐to‐measured output factors normalized to unity for a 10‐cm square field plotted as a function of equivalent square field size. Calculations use the PinEPID3 model and the models derived from it by either increasing or decreasing the modelling depth by 1 cm (PinEPID4 and PinEPID2 respectively) or by increasing or decreasing the machine energy by 0.2 MeV (PinEPID3+ and PinEPID3– respectively).

Once the depth and energy of the PinEPID3 model were selected, the other model parameters were again iteratively adjusted to achieve the best fit to the measured profiles and output factors. The sensitivity of this model to the choice of depth and energy was evaluated by altering either the modeling depth by ±1cm or the energy by ±0.2MeV. The altered models were compared with the original in terms of how well they fit the measured profiles (Fig. 1) and output factors (Fig. 2).

**Figure 3:**
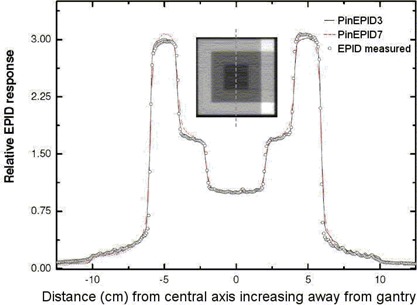
In‐plane profiles normalized to unity at YI=0, comparing the PinEPID3 and PinEPID7 model results with electronic portal imaging device (EPID) measurements for a well‐shaped intensity‐modulated radiation therapy test pattern.

**Figure 4:**
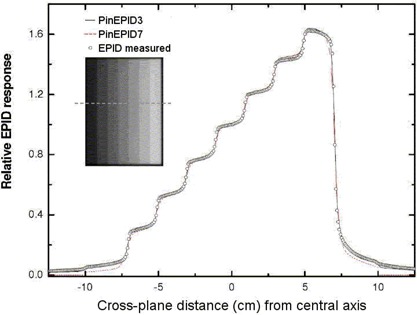
Cross‐plane profiles normalized to unity at XC=0, comparing PinEPID3 and PinEPID7 model results with electronic portal imaging devices (EPID) measurements for a step‐wedge‐shaped intensity‐modulated radiation therapy test pattern.

**Figure 5:**
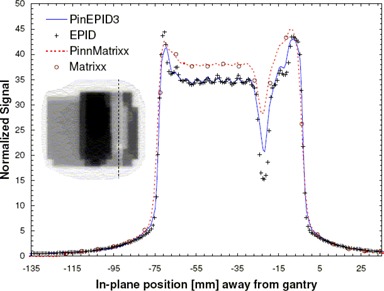
In‐plane profiles (normalized to unity at a point in the large dark region of the inset image) comparing measured with modeled data for beam 6 of the RPC phantom (Radiological Physics Center: M.D. Anderson. Houston, TX) plan. Measurements made with the I'mRT MatriXX (IBA Dosimetry, Schwarzenbruck, Germany) and with the electronic portal imaging device (EPID) are compared with planar dose calculations using the commissioned Pinnacle model and the Pinnacle EPID model respectively.

**Figure 6:**
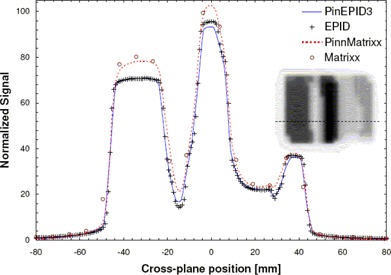
Cross‐plane profiles (arbitrarily normalized for best fit) comparing measured with modeled data for beam 9 of the RPC phantom (Radiological Physics Center: M.D. Anderson, Houston, TX) plan. Measurements made with the I'mRT MatriXX (IBA Dosimetry, Schwarzenbruck, Germany) and with the electronic portal imaging device (EPID) are compared with planar dose calculated with the commissioned Pinnacle model and the Pinnacle EPID model respectively.

The raw data saved by the imaging acquisition system represents the imager response per frame. This response may be calibrated to a dose if the number of acquired frames is known. This number can be determined by examining the image properties using Portal Vision tools. However, the number of frames could not be deduced from the raw data file alone during the post‐processing step. Each measured profile was therefore renormalized to fit the corresponding calculated profile at the central axis (Figs. 3 – 6). Absolute calibration of the EPID, while straightforward, has not been implemented in this work.

### C. Testing the EPID model

Step‐and‐shoot IMRT test patterns resembling a well, step‐wedge, tower, and checkerboard were planned and delivered with the EPID positioned at isocenter. For each field, the EPID was irradiated to 200 MUs with a dose rate of 300 MU/min. To calculate the EPID image, the clinical machine was replaced with the PinEPID3 or PinEPID7 machine model. This replacement must be done by running a UNIX script outside of the planning system so as to retain the planned leaf segments (which would otherwise be reset if the machine were to be changed within the plan).

To test the two models, planar doses were computed at a depth of 3 cm (97 cm SSD) and 7 cm (93 cm SSD) for each beam. The dimensions of the dose plane were chosen to match those of the portal images acquired at a plane intersecting the isocenter with the EPID: 512×384 pixels with 12.8 pixels per centimeter over an area of $40\times 30\;{\text{cm}}^{2}$. Figs. 3 and 4 show a relative comparison between measured EPID images and the planar dose maps computed using the EPID models for 2 of the 4 test patterns. A one‐dimensional chi‐square test was used to evaluate the model fit to the EPID measurements. The $\chi^{2}$ score was evaluated according to method described by Press et al.,^(31)^

(1) $$\chi^{2}=\sum_{i=1}^{512}\frac{{\left(EPID_{i}-PinEPID_{i}\right)}^{2}}{EPID_{i}},$$

where ${\text{EPID}}_{i}$ and ${\text{PinnEPID}}_{i}$ are, respectively, the values of the *i*th pixel of the acquired and calculated image normalized to the calculated value at the central axis.

### D. IMRT plan verification

A 9‐beam step‐and‐shoot IMRT treatment plan was designed for an RPC (Radiological Physics Center: M.D. Anderson, Houston, TX) head phantom with a 6 MV 21EX accelerator model in Pinnacle^3^ for the purpose of clinical trial credentialing. Before irradiation of the phantom, a two‐dimensional ion‐chamber array, I'mRT MatriXX (IBA Dosimetry, Schwarzenbruck, Germany), was irradiated at 100 cm source‐to‐axis distance with 5 cm polystyrene buildup. The dose for each beam was recorded and evaluated against the Pinnacle‐calculated planar dose map using the digital gamma evaluation algorithm within the OmniPro‐I'mRT software. The plan was also delivered at 300 MU/min to the EPID positioned at isocenter, and planar images were acquired for each beam separately. As described in the preceding subsection, the Varian 21EX machine model was replaced with the PinEPID3 machine model, and the planar dose maps were computed for each field at 3 cm depth (97 cm SSD) of water. Representative relative profiles are plotted in Figs. 5 and 6, which show the typical range of modulation found in IMRT fields. A relative comparison between the measured and computed images was done by normalizing the measured image to the calculated value at a point within a uniform high‐dose region of the field and then using a gamma evaluation technique implemented in‐house.^(32)^ For the gamma evaluation, a criterion of 3% of maximum dose difference and 3 mm distance to agreement was applied to generate the gamma map. The OmniPro I'mRT software was used to compare calculated and measured planar doses. Here, the digital gamma test was applied to all points registering greater than 10% of the maximum dose.

## III. RESULTS

### A. Model optimization

**Figure 7:**
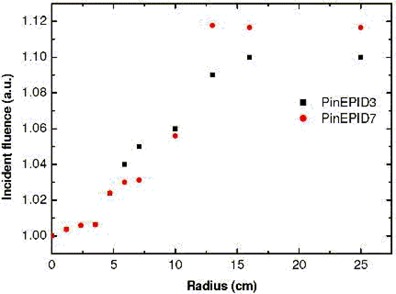
Relative incident photon fluence profile used for the PinEPID3 and PinEPID7 models, plotted in arbitrary units.

Certain parameters were straightforward to fit because they affected only certain aspects of the profile shape. Electron contamination was turned off, and the incident fluence profile was adjusted to match the in‐field portion of the 28‐cm square‐field profile derived from the EPID image. Incident fluence variation with off‐axis distance is plotted for both models in Fig. 7.

The primary source size has only a small effect on the slope of the penumbra, but it was minimized to achieve as steep a penumbra as possible. Jaw transmission was reduced relative to the clinical model to lower the calculated output factor for very small fields. Because most IMRT segments are collimated with MLC leaves, the leaf transmission was increased to boost the dose in the profile tails, and the leaf radius was slightly reduced to increase the rounding of the profile shoulder.

The energy and the secondary scatter source size and strength had the greatest effect on the fit of the model. Variation in any of these parameters affects profiles and output factors alike. The main challenge was to model the relatively large measured variation in phantom scatter factor (which suggests a depth of about 7 cm), while at the same time modeling the sharp drop‐off of dose out‐of‐field in the measured profiles (which suggests a much shallower depth). These two objectives tend to conflict. The PinEPID7 model (which was based on the clinical machine spectrum) yielded profiles with shoulders that were too rounded. Correcting for these shoulders by reducing the scatter source magnitude resulted in poor agreement with measured output factor variation with field size.

**Figure 8:**
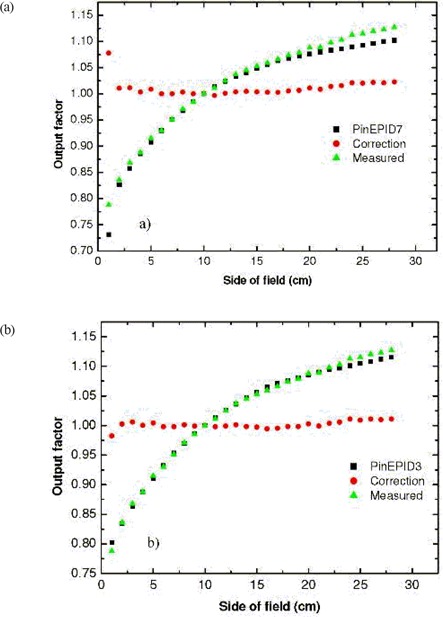
Output factors from the measured EPID image (filled triangles), and a PinEPID model computation (filled squares) plotted as a function of field size. The output correction factors, ${\text{OF}}_{c}$ (filled circles), are the ratios of measured‐to‐computed output factors; they equal unity in an ideal case. (a) Data for the PinEPID7 model. (b) Data for the PinEPID3 model.

The second model, PinEPID3, took advantage of shallow depth and lower energy to sharpen the profile penumbra for the same scatter source size and strength. A depth of 3 cm and energy of 0.8 MeV more closely fit the measured data. The selected energy is not truly an optimized value. The three parameters of energy, scatter source size, and scatter source strength are not independent with respect to their influence on profile and output factor variation. Starting with the scatter source parameters of the clinical machine model, the energy was adjusted to give the best agreement with the measured profile shoulders and output factors. The secondary source parameters were then fine‐tuned to get the best overall fit to the measured data. Agreement between measured and computed profiles with the PinnEPID3 model was slightly improved over the PinEPID7 model. The agreement between modeled and computed output factors was also improved for smaller fields (Fig. 8).

Other combinations of energy and depth may also result in acceptable models once secondary scatter source size and strength are optimized. However, at higher energy or shallower depth, the output variation with field size for larger fields tends to be less than that observed from measurement; at lower energies and greater depths, it tends to be greater than the variation observed from measurement. This differential is illustrated in Fig. 2, in which the modeling depth and energy of the PinEPID3 model are varied while the other modeling parameters are kept constant.

This variation also has an effect on the calculated profiles. Fig. 1 shows the percent error between computed and measured profiles of a 6×15−cm MLC collimated field normalized to the beam central axis. As in Fig. 2, the profiles are calculated using the PinEPID3 model or those derived from it by altering either the modeling depth by ±1cm (PinEPID2 and PinEPID4) or the incident photon energy by ±0.2MeV (PinEPID3+ and PinEPID3–). It is evident from Fig. 1 and observed in general that the models corresponding to shallower depths or higher energy have profile shoulders that are too square just within the field edge with dose falling too quickly outside the field. On the other hand, the models corresponding to greater depth or lower energy have profile shoulders that are too rounded within the field with tails that do not fall rapidly enough just outside the field edge. Note that, although the absolute error is large near the field edge at 7.5 cm, the distance to agreement is within 2 mm for all points in the high gradient region of the profile. The largest error (about 2%) occurs in the region of the profile under the MLC. For the PinEPID3 model, the maximum in‐field error is less than 1.3% at 7.1 cm from the central axis. Beyond that point, the gradient becomes steep, resulting in a maximum distance to agreement of about 1 mm. Under the MLC, the profiles agree to within about 1.5%; under the jaws, the agreement is within about 2%. This level of agreement is consistent for all of the profiles used to develop the model, provided that the measured profiles are symmetric about the central axis.

Table 2 summarizes the parameters that were found to best fit the measured profile and relative output factor data for the both PinEPID3 and PinnEPID7 machine models.

**Table 2:** Pinnacle^3^ optimized 6 MV parameters for a Varian 21EX linear accelerator^a^ (21EX), an electronic portal imaging device (EPID) model for 7 cm depth (PinEPID7), and an EPID model for 3 cm depth (PinEPID3)

*Parameters*	*21 EX*	*PinEPID7*	*PinEPID3*
Source size			
X (cm)	0.04	0.01	0.01
Y (cm)	0.04	0.01	0.01
Secondary source			
Gaussian height (cm)	0.08	0.1	0.1
Gaussian width (cm)	1.4	1.7	1.7
Jaw transmission	0.007	0.0011	0.002
MLC transmission	0.015	0.015	0.018
Rounded leaf tip radius (cm)	6.5	6	6
Energy	Modified	Modified	0.8 MeV
	Mohan spectrum	Mohan spectrum	

a Varian Medical Systems, Palo Alto, CA.

### B. Model evaluation

The profiles computed from the PinEPID7 model agreed with the measured EPID profiles to just within the VanDyk^(33)^ criterion (which is commonly used to evaluate a treatment planning model); those computed from the PinEPID3 model passed the criterion somewhat more easily. As shown in Fig. 8(a), computed output factors of the PinEPID7 model deviated from measurements by up to 7% for the smallest field, but otherwise the agreement was within about 2%. The output factors computed from the PinEPID3 model [Fig. 8(b)] agreed with those measured to within 2% for all field sizes.

Figs. 3 and 4 show relative EPID response profiles computed using the two EPID models PinEPID3 and PinEPID7 plotted with the EPID measurements for 2 of the 4 IMRT test patterns. In all 4 IMRT test patterns, both models fulfill the gamma criterion; however, as indicated in Table 3, higher $\chi^{2}$ scores resulted for PinEPID7 than for PinEPID3. The superiority of PinEPID3 resulted in subsequent comparisons considering only the latter model.

**Table 3:** Comparison of $\chi^{2}$ scores corresponding to the fit of the PinEPID7 and PinEPID3 models to electronic portal imaging device (EPID) measurements for each of the four intensity‐modulated radiation therapy test patterns analyzed

*Test pattern*	*PinEPID3*	*PinEPID7*
Tower	1.1	3.1
Well	3.0	5.1
Step‐wedge	1.1	8.1
Checkerboard	3.6	9.0

**Figure 9:**
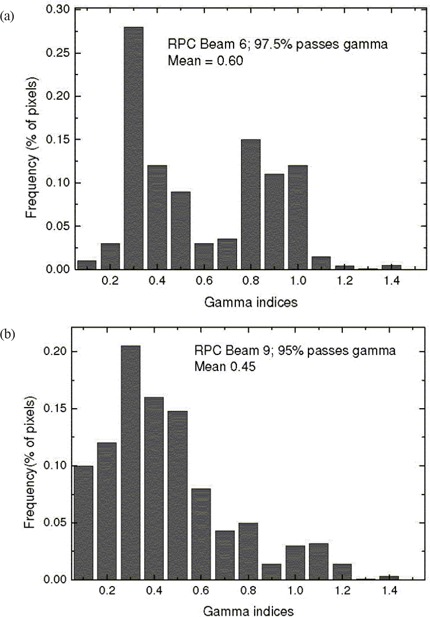
Histograms of gamma indices for (a) beam 6 and (b) beam 9 of the RPC head phantom (Radiological Physics Center: M.D. Anderson, Houston, TX). A criterion of 3 mm distance to agreement and dose difference of 3% of maximum dose was used in the evaluation procedure.

A 9‐beam IMRT plan for a RPC head phantom was verified using the process set out in Section II.D. Representative profiles plotted in Figs. 5 and 6 for 2 of the beams show good agreement with the EPID measurements. The gamma evaluation histograms calculated for all 9 beams indicate that 95% of all points pass the preset criterion of 3% dose or 3 mm distance to agreement for each beam. Fig. 9 shows representative histograms for beams 6 and 9. For comparison to I'mRT MatriXX results, the data were reanalyzed using the OmniPro software with the same dose and distance‐to‐agreement criterion, but with a 10% of maximum dose threshold. By selectively renormalizing the data, the percent of pixels passing the digital gamma test for beam 6 was 99.4 for the EPID as compared with 99.1 for the MatriXX, and the percent for beam 9 was 99.5 for the EPID as compared with 100 for the MatriXX.

## IV. DISCUSSION

A limitation of this technique for QA of IMRT fields is that it does not verify the clinical calculation, because the clinical machine is substituted by the EPID machine. This technique verifies errors in delivery only; other means are required for verifying the calculation.

With regard to the FF correction, our version of Portal Vision did not include a dosimetric workspace in which the beam profile correction is automatically applied. This step could have been performed as part of our own image processing, but we chose instead to model the flattened beam profiles recorded by the EPID, which does not contain horns because of the auto FF correction.

Although the physical characteristics of the EPID are well understood, they could not be directly incorporated into our model, which treats the EPID as water‐equivalent. At all but the shallowest depths, the falloff in the open beam EPID profile is more rapid than that in a clinical beam in water. At shallow depths, however, the shoulders of the EPID profile are more rounded than those of the clinical beam. The sharper profile falloff for the EPID as compared with an ionization chamber in water is partly a result of the higher aS500 EPID pixel resolution (0.781 mm per pixel as compared with a typical ion chamber diameter of about 4 mm) and partly a result of the smaller physical depth for the given water‐equivalent depth of 8 mm (specified by the vendor). The higher tails arise most probably because of over‐response to low‐energy photon scatter in the EPID. Other authors^(14)^ have shown that the rapid falloff in the penumbra region of the profiles is a result of the difference between the scatter kernel of water and that of the EPID and to the glare kernel of the EPID. The higher resolution of the aS500 as compared with an ion chamber is advantageous for measuring doses in sharp dose gradients and in penumbra regions because its small size means that it is not prone to partial‐volume effects.

The scatter characteristics of an EPID as compared with water for a 6 MV beam also influence output factor variation with field size. Matching computed and measured output factors is important, because Pinnacle^3^ applies a single equivalent square–based output factor correction, ${\text{OF}}_{c}$, calculated from the ratio of computed to measured output factors. The ${\text{OF}}_{c}$ depends only on the fixed jaw positions and not on the MLC positions, which are varied during segmented IMRT delivery, causing a corresponding variation in phantom scatter factor. For the PinnEPID3 model, ${\text{OF}}_{c}$ was less than 1% for all clinically relevant fields [Fig. 8(b)], recognizing that, in practice, segments of equivalent squares less than 3 cm or greater than 22 cm are disallowed. The small magnitude of this correction indicates that the phantom scatter factor variation with MLC collimated field size is accounted for in the model to within about 1%.

Rather than correct the EPID image with the beam profile, we used a radial photon fluence correction (plotted in Fig. 7). This approach necessitates the alignment of the beam central axis with the EPID center when imaging (to within a few millimeters) to reproduce the geometry when FF was acquired. The radial fluence correction plays the same role as the radial dose profile correction commonly used by others.

The modest improvement seen with the PinEPID3 model over the PinEPID7 model suggests that any number of assumed depths can lead to acceptable models once model parameters are suitably adjusted. The limited success of the PinEPID7 model is likely a result of 7 cm being too deep. However, no attempt was made to adjust the energy spectrum for this model, and so the possibility of improving the fit by that means cannot be excluded. On the other hand, PinEPID3 was developed by first choosing a reasonable modeling depth and then adjusting the EPID machine modeling parameters to obtain the best fit with the measured data. The alternative approach of starting with the clinical machine's modeled photon spectrum and then adjusting the modeling depth and other parameters is logistically more cumbersome to implement because modeling depth is not a parameter that is easily adjusted. As shown in Figs. 1 and 2, varying either the incident energy or the modeling depth alone in the PinEPID3 model does not improve the fit to the measured data. The profile error plot of Fig. 1 corresponding to the PinEPID3 model is consistent with generally observed agreement within 1.5% or 1 mm for in‐field points of measured profiles that are symmetric about the central axis. In regions outside the field, agreement with measured profiles was generally within 2%. Measured profiles that displayed asymmetry could not be modelled by any configuration of machine parameters in Pinnacle^3^, which requires that commissioning profiles be symmetric. The observed asymmetry in some profiles is attributed to backscatter from the support arm, which differs from the FF backscatter. It may be possible to reproduce the observed asymmetric profiles by combining the Pinnacle^3^ EPID machine model with a suitable choice of calculation geometry, but further work is needed to investigate this possibility.

Because the EPID is modelled at a single fixed depth for use in a fixed geometry, it is not necessary to model the variation in photon spectrum with depth. A monoenergetic photon spectrum was used, in which the energy was treated as a modelling parameter. The differential EPID response to beam hardening through MLC leaves was accounted for by adjusting the MLC transmission to fit the profile tails of MLC‐collimated fields. The influence of off‐axis beam softening on EPID response was accounted for by fitting the radial fluence (Fig. 7) to the in‐field portion of the EPID measured profiles.

Comparison of computed and measured profiles for geometric IMRT test shapes showed reasonably good agreement (within 2% of maximum dose in low gradient regions). Examples are illustrated in Figs. 3 and 4. Comparing the two machine models on the basis of a $\chi^{2}$ test confirms that the PinEPID3 model provides a better fit. This test was used to evaluate the models because it removes the biasing of data in the high‐dose region, penumbra, and tails by normalizing to local values. The poorer fit of the PinEPID7 model in the low‐dose tails of the test shapes bears out this conclusion. The largest discrepancy appears in Fig. 3, where asymmetry is observed in the measured EPID profile. This asymmetry is attributed to non‐uniform backscatter from the support arm of the EPID, which affects the FF pixel sensitivity calibration. This seems to be a persistent problem that becomes more severe as fields differ markedly in shape from the FF. Using the Pinnacle^3^ EPID model in conjunction with a specific phantom data set that models the effect of the support structures is a potential solution that warrants further investigation.

Applying the method of Section II.D for IMRT plan QA to the RPC phantom treatment plan, the PinEPID3 model generated profiles that generally overlaid the measurements for all 9 beams. The largest discrepancy occurred at narrow dose peaks and valleys where the Pinnacle model consistently under‐represents the extent of the signal excursions. Note, however, that these discrepancies are within 3% of the maximum dose except for the deep valley in Fig. 4, where the discrepancy is about 3%. An in‐house gamma analysis based on 3% maximum dose or 3 mm distance to agreement indicates that more than 95% of points pass for each beam. Re‐analysis of these beams using the digital gamma function of the OmniPro I'mRT software, rejecting pixels less than 10% of the maximum dose and selectively renormalizing the images, yields somewhat better results in which typically more than 99% of pixels pass. When this same analysis is applied to measurements using the I'mRT MatriXX with corresponding planar dose maps calculated in Pinnacle, a pass rate of more than 99% is typically seen for a 2% maximum dose or 2 mm distance‐to‐agreement digital gamma test. For these fields, the Pinnacle EPID model does not reliably predict the EPID response to better than 3%. Nevertheless, the higher resolution of the EPID relative to the MatriXX has an obvious benefit in terms of the size of errors that can be resolved. The failure of the EPID model to accurately reproduce the sharp EPID signal excursions suggests that the choice of a 3‐cm modeling depth allows for too much scatter, which broadens and shortens the dose peaks and valleys. The trade‐off in going to a shallower modelling depth is an insensitivity to scatter that is needed to model the variation of EPID response with field size. The compromise struck with the current model appears to work well with gamma analysis based on 3% maximum dose or 3 mm distance to agreement.

The current method may be used to replace film‐based IMRT QA by applying the schema set out in Section II.D. The number of IMRT plans undergoing QA verification at our center would make a film‐based system prohibitively labor‐intensive. Our IMRT QA program is currently based on ion‐chamber array measurements, but the EPID‐based approach presented here may find a role as a cost‐effective backup system because no additional infrastructure is needed. The method is simple to implement in any clinic with an IMRT‐capable treatment planning system and an EPID that can integrate over the entire radiation dose to be evaluated. In centers that have no EPID capability, the method may potentially be generalized to model any suitable portal imaging modality (such as film or computed radiography cassettes).

## V. CONCLUSIONS

A machine model created using Pinnacle^3^ physics tools can be used to calculate planar dose maps that approximate images acquired with the Varian aS500 EPID. Although the calculation assumes a water‐equivalent phantom, the modelled machine parameters are altered from those of the clinical machine to accommodate the portal imager's over‐response to low‐energy scatter (which contributes to a large variation of relative output with field size) and shallow effective depth (which gives rise to sharp profile penumbra). From this perspective, an assumed energy of 0.8 MeV and a depth of 3 cm may be considered a reasonable starting point for the model. Energy and secondary scatter source size and strength had the largest effect on profile penumbra and output factor, but their effects were not independent. These parameters were adjusted iteratively to best fit the data. Fitting the remaining parameters was straightforward. The resulting model predicts relative EPID response to within a few percent when small IMRT fields are test‐imaged. Further work is needed to account for backscatter from the support arm in the Pinnacle^3^ calculation. Nevertheless, our method is expected to find application as a backup system for pre‐treatment patient‐specific QA in our clinical IMRT program.

## ACKNOWLEDGMENT

The work reported here was carried out at the Juravinski Cancer Center in Hamilton Ontario, Canada—a Cancer Care Ontario partner.

## References

[1] Chang J , Ling CC . Using the frame averaging of aS500 EPID for IMRT verification. J Appl Clin Med Phys. 2003;4(4):287–299. 14604418 10.1120/jacmp.v4i4.2499 PMC5724460

[2] Talamonti C , Casati M , Bucciolini M . Pretreatment verification of IMRT absolute dose distributions using a commercial a‐Si EPID. Med Phys. 2006;33(11):4367–4378. 17153415 10.1118/1.2357834

[3] Van Esch A , Depuydt T , Huyskens DP . The use of an aSi‐based EPID for routine absolute dosimetric pre‐treatment verification of dynamic IMRT fields. Radiother Oncol. 2004;71(2):223–234. 15110457 10.1016/j.radonc.2004.02.018

[4] Wendling M , Louwe RJ , McDermott LN , Sonke JJ , van Herk M , Mijnheer BJ . Accurate two‐dimensional IMRT verification using a back‐projection EPID dosimetry method. Med Phys. 2006;33(2):259–273. 16532930 10.1118/1.2147744

[5] Bedford JL , Childs PJ , Nordmark Hansen V , Mosleh–Shirazi MA , Verhaegen F , Warrington AP . Commissioning and quality assurance of the Pinnacle^3^ radiotherapy treatment planning system for external beam photons. Br J Radiol. 2003;76(903):163–176. 12684232 10.1259/bjr/42085182

[6] Boellaard R , Essers M , van Herk M , Mijnheer BJ . New method to obtain the midplane dose using portal in vivo dosimetry. Int J Radiat Oncol Biol Phys. 1998;41(2):465–474. 9607366 10.1016/s0360-3016(98)00048-0

[7] Menon GV , Sloboda RS . Quality assurance measurements of a‐Si EPID performance. Med Dosim. 2004;29(1):11–17. 15023388 10.1016/j.meddos.2003.09.002

[8] Greer PB . Correction of pixel sensitivity variation and off‐axis response for amorphous silicon EPID dosimetry. Med Phys. 2005;32(12):3558–3568. 16475754 10.1118/1.2128498

[9] Moran JM , Roberts DA , Nurushev TS , Antonuk LE , El‐Mohri Y , Fraass BA . An active matrix flat panel dosimeter (AMFPD) for in‐phantom dosimetric measurements. Med Phys. 2005;32(2):466–472. 15789593 10.1118/1.1855012

[10] El‐Mohri Y , Antonuk LE , Yorkston J , et al. Relative dosimetry using active matrix flat‐panel imager (AMFPI) technology. Med Phys. 1999;26(8):1530–1541. 10501053 10.1118/1.598649

[11] Greer PB , Popescu CC . Dosimetric properties of an amorphous silicon electronic portal imaging device for verification of dynamic intensity modulated radiation therapy. Med Phys. 2003;30(7):1618–1627. 12906179 10.1118/1.1582469

[12] McCurdy BM , Luchka K , Pistorius S . Dosimetric investigation and portal dose image prediction using an amorphous silicon electronic portal imaging device. Med Phys. 2001;28(6):911–924. 11439488 10.1118/1.1374244

[13] Munro P , Bouius DC . X‐Ray quantum limited portal imaging using amorphous silicon flat‐panel arrays. Med Phys. 1998;25(5):689–702. 9608480 10.1118/1.598252

[14] Kirkby C , Sloboda R . Comprehensive Monte Carlo calculation of the point spread function for a commercial a‐Si EPID. Med Phys. 2005;32(4):1115–1127. 15895596 10.1118/1.1869072

[15] Warkentin B , Steciw S , Rathee S , Fallone BG . Dosimetric IMRT verification with a flat‐panel EPID. Med Phys. 2003;30(12):3143–3155. 14713081 10.1118/1.1625440

[16] Yeboah C , Pistorius S . Monte Carlo studies of the exit photon spectra and dose to a metal/phosphor portal imaging screen. Med Phys. 2000;27(2):330–339. 10718136 10.1118/1.598835

[17] Partridge M , Groh B , Spies L , Hesse BM , Bortfield T . A study of the spectral response of portal imaging detectors. Nuclear Science Symposium Conference Record, 2000 IEEE. 2000;3(2000):19/23–19/27.

[18] Kirkby C , Sloboda R . Consequences of the spectral response of an a‐Si EPID and implications for dosimetric calibration. Med Phys. 2005;32(8):2649–2658. 16193795 10.1118/1.1984335

[19] Greer PB , Vial P , Oliver L , Baldock C . Experimental investigation of the response of an amorphous silicon EPID to intensity modulated radiotherapy beams. Med Phys. 2007;34(11):4389–4398. 18072504 10.1118/1.2789406

[20] Ko L , Kim JO , Siebers JV . Investigation of the optimal backscatter for an aSi electronic portal imaging device. Phys Med Biol. 2004;49(9):1723–1738. 15152927 10.1088/0031-9155/49/9/010

[21] Siebers JV , Kim JO , Ko L , Keall PJ , Mohan R . Monte Carlo computation of dosimetric amorphous silicon electronic portal images. Med Phys. 2004;31(7):2135–2146. 15305468 10.1118/1.1764392

[22] Chen J , Chuang CF , Morin O , Aubin M , Pouliot J . Calibration of an amorphous‐silicon flat panel portal imager for exit‐beam dosimetry. Med Phys. 2006;33(3):584–594. 16878562 10.1118/1.2168294

[23] McCurdy BM , Pistorius S . A two‐step algorithm for predicting portal dose images in arbitrary detectors. Med Phys. 2000;27(9):2109–2116. 11011740 10.1118/1.1289375

[24] Steciw S , Warkentin B , Rathee S , Fallone BG . Three‐dimensional IMRT verification with a flat‐panel EPID. Med Phys. 2005;32(2):600–612. 15789607 10.1118/1.1843471

[25] McDermott LN , Louwe RJ , Sonke JJ , van Herk MB , Mijnheer BJ . Dose‐response and ghosting effects of an amorphous silicon electronic portal imaging device. Med Phys. 2004;31(2):285–295. 15000614 10.1118/1.1637969

[26] McDermott LN , Nijsten SM , Sonke JJ , Partridge M , van Herk M , Mijnheer BJ . Comparison of ghosting effects for three commercial a‐Si EPIDs. Med Phys. 2006;33(7):2448–2451. 16898447 10.1118/1.2207318

[27] Chang J , Mageras GS , Chui CS , Ling CC , Lutz W . Relative profile and dose verification of intensity‐modulated radiation therapy. Int J Radiat Oncol Biol Phys. 2000;47(1):231–240. 10758329 10.1016/s0360-3016(99)00555-6

[28] Mackie TR , Scrimger JW , Battista JJ . A convolution method of calculating dose for 15‐MV X‐rays. Med Phys. 1985;12(2):188–196. 4000075 10.1118/1.595774

[29] Mackie TR , Ahnesjo A , Dickof P , Snider A . Development of a convolution/superposition method for photon beams. In: Proceedings of the 9th International Conference on the Use of Computers in Radiation Therapy; The Hague, Nehterlands; 22–25 June 1987. Amsterdam: Elsevier Science; 1987:107–110.

[30] Papanikolaou N , Mackie TR , Meger–Wells C , Gehring M , Reckwerdt P . Investigation of the convolution method for polyenergetic spectra. Med Phys. 1993;20(5):1327–1336. 8289713 10.1118/1.597154

[31] Press WH , Teukolsky SA , Vetterling WT , Flannery BP . Numerical recipes in C: the art of scientific computing. 2nd edition New York (NY): Cambridge University Press; 1992 1256 p.

[32] Low DA , Dempsey JF . Evaluation of the gamma dose distribution comparison method. Med Phys. 2003;30(9):2455–2464. 14528967 10.1118/1.1598711

[33] Van Dyk J , Barnett RB , Cygler JE , Shragge PC . Commissioning and quality assurance of treatment planning computers. Int J Radiat Oncol Biol Phys. 1993;26(2):261–273. 8491684 10.1016/0360-3016(93)90206-b

